# Systematic Review of Level 1 and Level 2 Screening Tools for Autism Spectrum Disorders in Toddlers

**DOI:** 10.3390/brainsci10030180

**Published:** 2020-03-19

**Authors:** Serena Petrocchi, Annalisa Levante, Flavia Lecciso

**Affiliations:** 1 Institute of Communication and Health, Università della Svizzera Italiana, Via Buffi 13, 6900 Lugano, Switzerland; 2 Lab of Applied Psychology and Intervention, Department of History, Society and Human Studies, University of Salento, Via di Valesio, 73100 Lecce, Italy; annalisa.levante@unisalento.it (A.L.); flavia.lecciso@unisalento.it (F.L.); 3 Applied Research Division for Cognitive and Psychological Science, IRCCS European Institute of Oncology, Via Ripamonti 435, 20141 Milano, Italy; 4 Department of History, Society and Human Studies, University of Salento, Via di Valesio, 73100 Lecce, Italy

**Keywords:** autism, level 1 and level 2 screening tools, systematic review, COSMIN, PRISMA

## Abstract

The present study provides a systematic review of level 1 and level 2 screening tools for the early detection of autism under 24 months of age and an evaluation of the psychometric and measurement properties of their studies. Methods: Seven databases (e.g., Scopus, EBSCOhost Research Database) were screened and experts in the autism spectrum disorders (ASD) field were questioned; Preferred Reporting Items for Systematic review and Meta-Analysis (PRISMA) guidelines and Consensus-based Standard for the selection of health Measurement INstruments (COSMIN) checklist were applied. Results: the study included 52 papers and 16 measures; most of them were questionnaires, and the Modified-CHecklist for Autism in Toddler (M-CHAT) was the most extensively tested. The measures’ strengths (analytical evaluation of methodological quality according to COSMIN) and limitations (in term of Negative Predictive Value, Positive Predictive Value, sensitivity, and specificity) were described; the quality of the studies, assessed with the application of the COSMIN checklist, highlighted the necessity of further validation studies for all the measures. According to COSMIN results, the M-CHAT, First Years Inventory (FYI), and Quantitative-CHecklist for Autism in Toddler (Q-CHAT) seem to be promising measures that may be applied systematically by health professionals in the future.

## 1. Introduction

Recently, U.S. data showed that the median age at earliest Autism Spectrum Disorders (ASD; [1]) diagnosis ranged from 28 to 39 months for children aged 4 [2] and is 40 months for children aged 8 [3]. According to these data, a screening procedure during the regular well-baby check-ups was recommended [2,4] with the aim to detect the warning signs of ASD (e.g., precursors of Theory of Mind; [5]). As suggested by several authors [6,7], the process should involve the early screening of warning signs and the subsequent diagnosis made through clinical judgement, in combination with the application of reliable and standardized gold-standard measures (e.g., the Autism Diagnostic Interview-Revised, [8]; the Autism Diagnostic Observative Schedule-2, [9]).

Earlier diagnosis of ASD could lead to earlier intervention for children, which could enhance their adaptation [10,11,12] or improve their social competence (e.g., emotional expression; see for details [13,14,15], prevent secondary developmental disturbances [16], and lead to better outcomes [17,18,19]. Screening measures that are suitable for use in young children (i.e., less than 24 months) are available, and can be classified as either Level 1 or Level 2 instruments [19]. Level 1 screening measures have been developed for the general population (unselected population) to identify children at risk of developmental disorders, including ASD. Level 2 screening tools have been developed to identify children at risk of ASD either because they are already under observation for developmental concerns, or because they failed Level 1 screening, or because they are siblings of children with ASD. The latter, as demonstrated for example by Lauritsen and colleagues [20], have a strong genetic risk. As Robins and Dumont-Mathieu [19] noted, several measures, developed for level 1 or level 2 screening, have been applied to other populations, determining a “hybrid” application of them. The present systematic review focuses on level 1 and level 2 screening measures of ASD, which can be administered to GPs and/or to parents or other professional groups (e.g., nurses, social workers).

In the last few years, eight reviews [21,22,23,24,25,26,27,28] have examined measures for the early detection of risk of ASD. Daniels and colleagues [21] focused on studies investigating approaches aiming at improving the early detection of ASD. This was a systematic review using five databases, although the authors chose to include studies limited to the United States. Garcia-Primo and colleagues [22] and Sappok and colleagues [24] conducted non-systematic reviews considering both measures for the early detection of risk for ASD and for diagnosis. The study by Garcia-Primo and colleagues [22] was limited to measures applied in Europe, published up to 2012, and the search was restricted to two databases (PubMED and PsycINFO). The review by Sappok and colleagues [24] was limited to one database (PubMED) and it considered measures developed for German and English speakers. Zwaigenbaum and colleagues’ review [25] was limited to one database (PubMED) and the research strategy included papers published up to 2013. McPheeters and colleagues [23] made a valuable systematic review of the ASD screening tools for children who were referred for developmental disorders other than ASD and were under 36 months old.

Nevertheless, their search strategy included four databases and they considered studies published up to 2000. Marlow and colleagues [26] carried out a systematic review extracted data from four databases and included papers published up to 2017. The meta-analysis by Sánchez-García and colleagues [27] evaluated the accuracy of screening measures according only to their sensitivity, specificity, positive, and negative predictive values (PPV and NPV respectively); furthermore, their electronic search was limited to 5 databases and included paper published up to 2015. Finally, the review by Thabtah and Peebles [28] provided a no systematic review on screening tools administrable from toddlerhood to adulthood, but the authors did not report the search strategy (i.e., databased searched; range of publication years considered) applied and described the tools only in terms of sensitivity and specificity.

Summarizing, most of the above-mentioned reviews are not systematic [22,24,25,28], have limited search strategies to 1–5 databases [22,23,24,26,27], or focus on a specific geographic area as Europe or USA [21,22,24]. Furthermore, they did not analyze the psychometric and measurement properties of the measures with the exception of Sánchez- García and colleagues [27] meta-analysis which applied the Bayesian Hierarchical Model to evaluate some psychometric properties associated to accuracy. Overall, researchers cannot derive considerations regarding the methodological quality of the studies.

To overcome the limitations of the previous reviews, we provided a systematic search on level 1 and 2 screening tools for ASD and an evaluation of their psychometric properties according to the COSMIN checklist [29,30]. The COSMIN checklist is a ‘standardized tool for assessing the methodological quality of studies on measurement properties’ [31] developed based on a Delphi study which is a standardized.

The specific research questions were: (RQ1) What are the level 1 and level 2 screening measures to detect early signs of risk of ASD in children under 24 months of age? (RQ2) What are the psychometric properties of the studies of Level 1 and Level 2 measures and what is their quality evaluated applying the COSMIN checklist? (RQ3) Is there one (or more) promising instrument(s) for the early detection of risk of ASD according to COSMIN results?

To give the reader a full and comprehensive view of the characteristics of the Level 1 and Level 2 measures available, and since the COSMIN protocol evaluates the quality of the study, but not the quality of the tool, we collected data on sensitivity, specificity, PPV, and NPV for all the included measures and we provided a discussion about those properties.

## 2. Materials and Methods

The systematic review is based on a published protocol [32], in which the authors reported a comprehensive description of the steps to follow, the methodology, and the process of the review. Furthermore, the authors provided the format of the tables to be used for the main descriptive data of the papers included in the review and the results of the examination of the psychometric properties. The methodology applied was developed based on the Preferred Reporting Items for Systematic Review and Meta-Analysis (PRISMA) guidelines [33] for identifying the papers to be included in the review. An electronic search was conducted using PsychINFO, the Psychology and Behavioral Sciences Collection, Cumulative Index of Nursing and Allied Health Literature, Scopus, the Education Resources Information Center, Google Scholar, and Pubmed (including MEDical Literature Analysis and Retrieval System OnLINE). The keywords applied were: ‘early diagnosis or diagnos *’, ‘ASD screen *’, ‘ASD detect *’, ‘ASD or autism or autist *’, ‘assessment tool’, ‘surveillance’, ‘develop * surveillance’, ‘assess *’, ‘instrument *’, ‘measure *’, ‘psychometric properties’, ‘standardiz *’, ‘tool*’, and ‘validat *’. A secondary hand search was performed to include references and citations from the identified papers. The electronic search was carried out by an author who extracted the records and tabulated the references in an excel file. Two authors independently screened the records to exclude duplicates and to remove papers according to pre-defined inclusion/exclusion criteria. The two authors reported their decisions in two different excel files and they compared their findings record by record. In case of disagreement, a third author arbitrated. Finally, three clinicians and three research experts in ASD, working respectively for the Public Health Service and for Universities respectively, were questioned. Based on the inclusion/exclusion criteria, they did not suggest any other relevant existing measure/study different from those already included in the present review.

Predefined inclusion criteria were: (1) level 1 and level 2 screening measures of ASD for children under 24 months; (2) validation studies, standardization of measures, cross-cultural comparisons, longitudinal, or follow-up studies; (3) published papers in peer- reviewed journals; (4) papers written in English; and 6) a year of publication between 1990 and October 2019. Other reviews on the same topic were examined to extract citations of studies that were eligible for our final list. Furthermore, exclusion criteria were defined as following: (1) measures of the diagnosis of ASD; (2) retrospective studies and systematic reviews; (3) measures of risk detection/diagnosis of others developmental disorders; (4) procedures for the detection of ASD other than questionnaires, interviews and observation procedures (i.e., biological markers, fMRI, blood test); (5) epidemiological studies and guidelines for experts; (6) publications that are not in peer-reviewed journals; (7) papers without the specific aim to evaluate psychometric properties or validity properties of the measures; (8) dissertation thesis or conference papers.

The evaluation of the measures applied the COnsensus-based Standards for the selection of health Measurement INstrument (COSMIN) checklist [29,30,31]. The COSMIN checklist applies nine boxes identifying the main measurement properties: (A) internal consistency (i.e., the degree to which the items of a questionnaire correlate with each other and evaluate the same concept); (B) reliability (i.e., the ability to measure a construct over time or by different persons); (C) measurement error (i.e., the error of the score not attributed to true changes in the construct); (D) content validity (i.e., the degree to which the items reflect adequately the construct measured); (E) structural validity (i.e., evaluating whether the hypothesized latent factor(s) reaches a good fit of the data); (F) hypothesis testing (i.e., considering whether the construct measured by the questionnaire reaches the expected relations with other variables); (G) cross-cultural validity (i.e., giving information on the generalization properties of the measure when applied in a different cultural context); (H) criterion validity (i.e., the degree to which the measure correlates with a ‘gold-standard’ measure); and (I) responsiveness (i.e., evaluating whether the measure predicts a change over time). Each box contains a different number of items (ranging from 5 to 18) evaluating ‘design aspects and statistical methods’ of a study [31] (p. 651), which require a mandatory assessment to obtain a full appraisal of the properties.

The COSMIN checklist provides a multi-step evaluation. The first step concerns the decision about which measurement properties have been assessed in a target paper among the nine boxes, and it is achieved by applying a binary scale (i.e., present vs. absent) considering the whole paper. For example, if the internal consistency (i.e., box A) is a property evaluated in a paper, then ‘present’ is attributed to box A for that paper.

The second step refines the evaluation undertaken in step 1. For each box marked as ‘present’ in step 1, the evaluator works through the questions, assigning to each of them an evaluation on a dichotomous scale (‘yes’ if the specific properties suggested by the question are present or ‘no’ if the specific properties suggested by the question are not present).

Finally, in the third step, the score obtained in step 2 is further refined. Every item marked as ‘yes’ in the previous step is now evaluated on a four-point Likert scale: excellent (+++), good (++), moderate (+), or poor (0).

A final evaluation for each box is obtained by considering the lowest score attributed to that box according to the worst score counts [31] (p. 651) procedure. Therefore, if even only one item in the box obtained a poor score, the measurement property for that box is rated as poor. Two authors independently applied the COSMIN checklist on 20 papers with an inter-rater agreement of Cohen’s k = 0.94.

## 3. Results

### 3.1. Overview of the Studies and Measures

**Figure 1:**
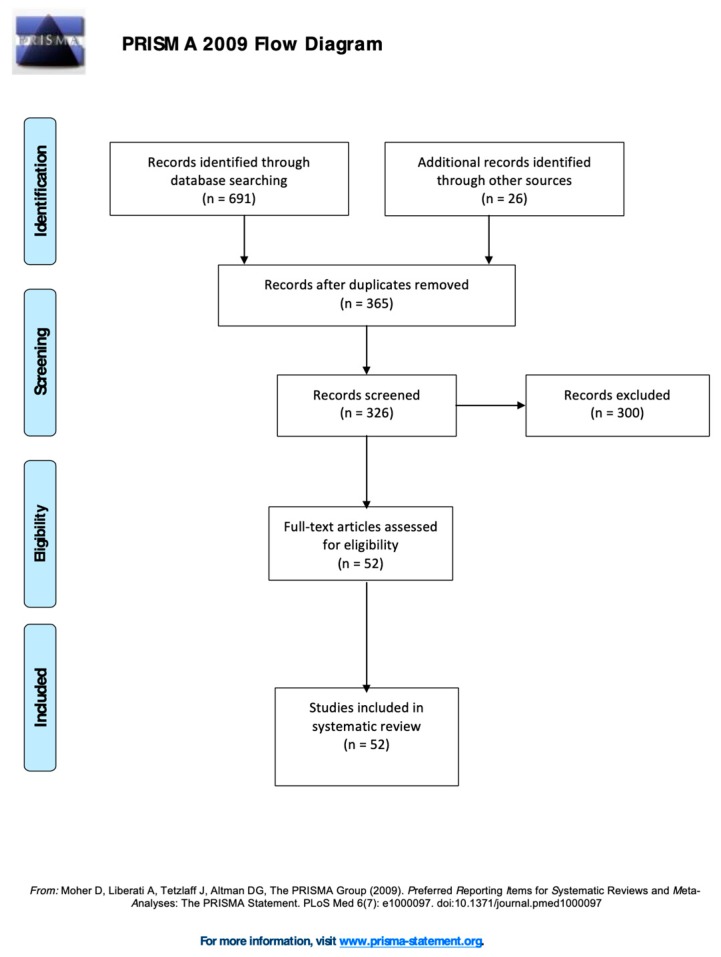
PRISMA flow diagram.

Figure 1 shows the PRISMA diagram.

The electronic search allowed to identify 691 records and a second-hand search added 26 more records. According to the inter-raters decision-making process, during the screening, two authors independently removed 365 duplicates and 300 papers according to the exclusion criteria. The final eligible number of papers included in the systematic review was 52 ([34,35,36,37,38,39,40,41,42,43,44,45,46,47,48,49,50,51,52,53,54,55,56,57,58,59,60,61,62,63,64,65,66,67,68,69,70,71,72,73,74,75,76,77,78,79,80,81,82,83,84,85]. The consistency between the two authors who screened these records was high (Cohen’s k = 0.89). Sixteen measures were evaluated and classified into 3 categories: observational checklists (*n* = 4), questionnaires (*n* = 10), and interviews (*n* = 2). Table 1 reports the general details of each measure.

Table 2 showed the details of the studies included in the systematic review. Specifically, we reported the measure name, authors and year of the study, the type of the design, population recruited, the application level (1, 2, or “hybrid”), and the diagnostic accuracy properties (i.e., sensitivity, specificity, PPV, NPV).

We found six level 1 measures (i.e., the Checklist for Early Signs of Developmental Disorders; the Early Screening of Autistic Traits Questionnaire; the First Year Inventor; the Joint Attention OBServation; the Screening for Infants with Developmental Deficits and/or Autism; the Young Autism and other developmental disorders CHeckup Tool: 18- month-olds’ version) administered to the general population retrieved in 6 longitudinal studies and 3 cross-sectional studies.

### 3.2. Overview of the Studies and Measures

The search strategy allowed to find four level 2 measures (i.e., the Autism Detection in Early Childhood; the Autism Observation Scale for Infants; the Baby and Infants Screen for Children with aUtIsm Traits; the Parent Observation of Early Markers Scale) that were also retrieved from the systematic search evaluated in eleven studies with a cross-sectional design and in two studies with a longitudinal design. Those measures were administered to two groups of children. The first group consisted of children who were already receiving attention from the local mental health service due to developmental concerns, children suspected of developmental delay, or children qualified for a medical condition that could determine a developmental delay including ASD comorbidity (i.e., epilepsy, hydrocephaly, Down’s syndrome, and cerebral palsy). Henceforth this group is identified as Developmental Concerns group (DC). The second group included twins or younger siblings of children with an ASD diagnosis, henceforth defined as Genetic Risk group (GR) because they have high probability to develop ASD [20]. The studies included in level 2 aimed either to: (a) test a screening measure on DC or GR groups; (b) compare DC and GR groups between them; (c) follow DC/GR group until the diagnosis; or, finally, (d) compare children from the general population to DC or GR groups.

Table 2 shows also the details of the six ‘hybrid’ measures (i.e., the CHecklist for Autism in Toddlers; the Developmental Behavior Checklist: Early Screen; the Modified Checklist for Autism in Toddlers; the Modified Checklist for Autism in Toddlers-Revised with Follow-up; the Quantitative- CHecklist for Autism in Toddlers; the Three-Item Direct Observation Screen) that were developed mainly for level 1 and/or level 2 screening, but they were also administered to clinical populations (i.e., children who had already received a diagnosis of ASD or of another developmental disorder). Those studies aimed either to: (a) apply the measure to a clinical sample, (b) compare samples with different diagnoses (ASD vs. PDD-NOS vs. ODD), or, finally, (c) compare children from the general population with children with an ASD diagnosis. Eleven studies were longitudinal and 19 had cross-sectional design.

The sensitivity, specificity, PPV, and NPV of the measures are extensively reported in the validation studies of the M-CHAT, M-CHAT R/F, and ADEC. For other measures (i.e., CESDD, JA-OBS, POEMS, DBC-ES, and TIDOS) there is only one study, each containing information of the NPV and PPV. All the other measures did not report any positive or negative predictive values. Overall considered, the measures for which the PPVs and NPVs were reported, demonstrated from moderate to high predictive values, although for the M-CHAT results can be considered more stable compared to other measures that need further and deeper exploration of these properties. Quality of assessment of the studies

Table 3 shows the results of the evaluation of each psychometric properties of the studies through the application of the COSMIN checklist. For each box, we reported a summary of the assigned scores.

The quality of assessment revealed a heterogeneous picture. Specifically, 24 studies out of 52 received an evaluation of the internal consistency (Box A) and the scores were fair or poor, with the exception of the studies on FYI and the Q-CHAT, which received excellent scores. The reliability (Box B) was evaluated in 17 studies and the majority of the scores rating from fair to poor. Only studies considering the CHAT and POEMS received respectively an excellent and good evaluation. The measurement error (Box C) was assessed in 5 longitudinal studies and received poor or fair evaluations.

The Box D (i.e., content validity) was evaluated in 9 studies and it received excellent evaluations for studies considering AOSI, BISCUIT, CHAT, FYI, M-CHAT, POEMS, Q-CHAT, SEEK, and TIDOS. Structural validity (Box E) was evaluated in 7 studies, but only 3 received excellent scores regarding two measures (i.e., M-CHAT and Q- CHAT). The Hypothesis testing (Box F) was evaluated for several studies, which received fair or poor scores, whereas those on FYI and the M-CHAT-R/F received good evaluations, and that on M-CHAT was evaluated as excellent. For the studies on JA-OBS, the SEEK, and the YATCH-18 the property was not evaluated.

The cross-cultural validity (Box G) was examined in 11 studies and received fair or poor scores. The box criterion validity (H) was evaluated for all studies, with the exception of the one on Q-CHAT and one on SEEK. This property was rated as excellent or good in four studies for four measures (FYI, M-CHAT, M-CHAT-R/F, and Q-CHAT); whereas for all other studies it was evaluated as fair or poor. Finally, the responsiveness (Box I) was the least-evaluated property with only 3 studies receiving scores from fair to poor.

As Table 3 shows the reasons leading to the attribution of fair and poor scores are above all the missing data and the sample size criteria and the fact that they are evaluated across several measurement properties. These criteria were evaluated by the COSMIN with a conservative approach [86], which will be discussed in the following section.

## 4. Discussion

The systematic review identified six level 1 measures and four level 2 measures. Moreover, the present systematic review found that six screening tools were applied to clinical populations. Among the variety of methodologies of the level 1 and level 2 measures, the questionnaire was the most applied due to several inherent advantages. First, questionnaires are normally administered in a very short time, do not require specific knowledge or training, and are much less invasive than observational checklists or interviews. Second, they often do not require specific training on the coding system or the interpretation of the scores. For many questionnaires, the imputation of a final score and the attribution of a meaning to it do not involve any clinical interpretation or specific knowledge of ASD. Nevertheless, questionnaires have several limitations. First, the score depends on the subjectivity of the informants. Since questionnaires are designed for parents, they could under- or overestimate the early signs of risk based on their ability to detect them and to distinguish signs of risk from normal deviation from the developmental trajectories. However, the impact of this limitation could be minimized with longitudinal studies testing and comparing the level 1 and level 2 screening instruments with the gold- standard measures (e.g., Autism Diagnostic Observation Schedule-2, [8]) for the diagnosis of ASD. Another inherent limitation of the questionnaires is social desirability bias in the form of over-reporting desirable behaviors. Future research in this field is needed to develop one or more validity scales, as for other clinical psychological testing procedures (i.e., the MMPI-2; see [87]).

The second aim of the present review was to evaluate the psychometric characteristics of the included measures following the COSMIN checklist. Two main considerations could be drawn by our results, one pertaining to the quantity of the psychometric evaluations and the other to their quality. First, it should be noticed that in the studies included in our systematic review, there are several psychometric properties more frequently evaluated than others. A high number of studies contained data that allowed the evaluation of the internal consistency, reliability, hypothesis testing, and criterion validity; whereas the measurement error, content validity, structural validity, cross-cultural validity, and responsiveness have been evaluated in a low number of studies. The second element to be considered is the quality of the evaluations themselves. Indeed, a high frequency of evaluations of a given property not always corresponds to a high quality of evaluation of that property. For example, the content validity was the property less frequently assessed, compared to the others, but it was rated as excellent for all the studies examined. On the other side, the hypothesis testing was frequently evaluated, but received poor or fair scores. These findings should give an impetus to researchers to design validation studies with a focus on both the quantity of the properties and their quality.

Considered overall, one very common problem for all the studies is the treatment of missing data. Few authors explicitly quantified the missing data in their data set, and very few explained the method that they followed to treat missing data. For studies that aim to identify early signs of risk of ASD, the treatment of the missing data represents a crucial aspect. For this specific case, the imputation of data through statistical procedures risks altering the data structure and the distribution beyond the over-/underestimation of the risk of ASD. Thus, it is quite important that, in the future, researchers explain whether and how they have treated missing data in their sample, especially for the parent-reported measures, for which it is more likely to have items with no answers.

According to the COSMIN evaluation, our findings highlight the necessity of further validation studies for all the measures included in the present review. Longitudinal studies involving general population following a sample over time with the purpose of making a diagnostic evaluation are particularly needed. This will allow for an in-depth study the psychometric properties, to compare the results from different measures, and consequently to increase their criterion validity, and specifically the sensitivity and the specificity through the comparisons with the gold standard measures.

Special consideration had to be drawn regarding the Sensitivity, Specificity, PPV, and NPV of the measures because they are not included in the COSMIN checklist. These properties are extensively reported in the validation studies of the M-CHAT, M-CHAT R/F, and ADEC. For other measures (i.e., CESDD, JA-OBS, POEMS, DBC-ES, and TIDOS) there is only one study each containing information of these properties (see Table 2 for the specific values). All the other measures did not report any positive or negative predictive values. Overall considered, the measures for which the Sensitivity, Specificity, PPVs and NPVs were reported, demonstrated from moderate to high predictive values (see also [27]), although for the M-CHAT results can be considered more stable compared to other measures that need further and deeper exploration of these properties.

The third and final research question aimed at the identification of one (or more) promising instrument(s) for the assessment of early signs of risk of ASD according to the COSMIN evaluations of the studies. We consider the questionnaires such as the FYI, the M-CHAT, and the Q-CHAT as promising screening measures because, according to the COSMIN evaluation, they have high number of psychometric properties evaluated and high methodological quality attributed to them. Although we found these measures promising, none of them can be currently considered as the gold standard in the early detection of risk of ASD and further development in this field is desirable. For example, future studies should improve sensitivity, specificity, NPV, and PPV properties of those measures since they are not considered at all for the FYI and they are barely considered for M-CHAT and Q-CHAT, as also suggested by [27].

On the contrary, the interviews and the observational checklists have both low number of validation studies (with the exception of the M-CHAT-R/F) and low methodological quality attributed to them. Further research should be developed on these methods of evaluation focusing on their psychometric properties, as it may be useful for health professionals to have a range of tools available for ASD risk detection that allows an in-depth analysis.

The present systematic review has several limitations. First, the COSMIN checklist is a standardized protocol for the assessment of the methodological quality of a study and not of the instrument itself. However, as suggested by others [see 86] the evaluation of the methodological quality of a study is the first step to determining whether its results are reliable and trustworthy. In other words, evaluating the methodological quality of a study allows to discover risk of bias in the results. Thus, the assessment of the quality of the study is directly related to the assessment of the measure administered in that study. Moreover, one of our inclusion criteria considered all the “validation studies, standardization of measures, cross-cultural comparisons, longitudinal, or follow-up studies”, which are studies evaluating measurement and validity properties of a screening measure. Therefore, we applied the COSMIN checklist to evaluate measurement properties of studies that, in turn, evaluate the measurement properties of the screening measures. Thus, the evaluation of the properties of a study, in this case, is a proxy of the evaluation of the measure validated in that study.

Second, the worse score counts policy of the COSMIN could lead to a negatively biased view of the measure. In this vein, the COSMIN itself explains that every item of its evaluation represents an important part of the overall assessment, so a poor rating for any item should be considered as a serious flaw. Furthermore, we would like to focus on the COSMIN evaluation of the sample size. According to [31], the sample size is evaluated as excellent when it is ≥ 100, as good when it ranges 50-99, fair when it ranges 30-49 is fair, and poor when it is < 30. This categorization is a good criterion when applied to the general population, while when risk and/or clinical groups are considered, the COSMIN sample evaluation should be carefully considered according to the prevalence rate of ASD. According to this premise, recently, the researcher who developed the COSMIN protocol reformulated the evaluation of the sample size (see [86]).

Third, the Sensitivity, Specificity, Positive Predictive Value (PPV) and the Negative Predictive Value (NPV) are not evaluated in the COSMIN checklist. Within the context of screening measures for ASD, it is important that professionals are confident when using a given tool. In this field, the predictive values provide valuable information on the probability of a tool to identify that people with high scores indeed have high risk (PPV) and, vice versa, that people with low score have low risk (NPV). To avoid the omission of such important information, we extracted values of the NPVs and PPVs from the studies, we reported them in Table 2 and we discussed the evidence.

Finally, like every systematic review, the definition of inclusion criteria could have limited the electronic search, and we could have omitted several studies.

The present systematic review has two main strengths. First, the review provides an updated and complete overview of the current level 1 and level 2 screening measures for ASD. Second, our findings provide researchers and clinicians (i.e., pediatricians, GP, psychologist) the analytical knowledge on psychometric properties of the measures through the evaluation of the methodological quality of their validation studies. The outcomes of the systematic search and the results of the evaluation of the psychometric properties, through the application of the COSMIN criteria, may guide researchers and clinicians in their selection of one (or more) instrument(s), according to their specific purposes. A critical and reasoned choice of a measure combined with the good communication between clinical and patients [88] could allow for defining systematic screening procedure on general population. This is the first step for early identification of risk of ASD, which, in turn, may lead to a timely diagnosis and ultimately to better outcomes for children [10,17,18] and families [89].

## Figures and Tables

**Table 1:** Descriptive details of Level 1 and 2 screening tools include in systematic review.

Measure Name (Short Name)	Short Description of the Dimension(s) Measured	Admin. Age (Months)	Number of Items	Type of Answer	Admin. Time (Minute)	Admin. Method	Cut-Off	*N*° of Validation Studies Included
**Autism Detection in Early Childhood (ADEC)**	Social interaction behaviors and social communication behaviors.	12–36	16	3-point Likert scale	10	Observational checklist for professionals	11	4
**Autism Observation Scale for Infants (AOSI)**	Social communication behaviors, non- social behaviors,	6–18	18	3-point Likert scale	15-20	Observational checklist for professionals	n.s.	3
**Baby and Infants Screen for Children with aUtIsm Traits (BISCUIT)**	Part 1 ASD symptoms; Part 2 comorbid psychopathology; Part 3 behavioral problems.	17–37	Part 1: 62; Part 2: 65; Part 3: 17.	3-point Likert scale	15	Parent-interview	Part 1: 17; Part 2: 39; Part 3: 17.	5
**Checklist for Early Signs of Developmental Disorders**	Language and social functioning.	3–39	25	Yes/No	Not declared	Parent-reported questionnaire	2	1
**CHecklist for Autism in Toddlers (CHAT)**	Social play, social interest, pretend play, joint-attention, proto-declarative pointing, imitation; B functional play, proto-imperative pointing, motor development, rough and tumble play.	18	Part A: 9; Part B: 5	Yes/No	15	Part A: parent- reported questionnaire; Part B: professionals- reported questionnaire	3 key item	2
**Developmental Behavior Checklist: Early Screen (DBC-ES)**	Social, verbal, and non-verbal communication, restricted and repetitive behaviors and interests	18–48	17	3-point Likert scale	5–10	Parent-reported questionnaire	11	1
**Early Screening of Autistic Traits Questionnaire (ESAT)**	Social- communication skills, stereotyped behaviors, reactions	14–15	14	Yes/No	5–10	Parent-reported questionnaire	3	2
**First Year Inventory (FYI)**	Social communication and sensory regulatory domains.	12	63	4 point Likert scale; multiple choice; two open-ended question.	10	Parent-reported questionnaire	30 (95th); 40 (98th); 50 (99th) [73] (Reznick et al., 2007); 22.55 (95th); 28.14 (98th) [39]; 19.2 (96th) [84].	3
**Joint Attention OBServation (JA- OBS)**	Joint attention	20–48	5	Yes/No	10	Observational checklist for professionals	2	1
**Modified Checklist for Autism in Toddlers (M- CHAT)**	Social interest, pretend and functional play, joint-attention, proto-declarative pointing, imitation, motor development, rough and tumble play.	16–30	23	Yes/No	5–10	Parent-reported questionnaire	2 for the critical items (2–7–9–13–14–15) or 3 for the total score	13
**Modified Checklist for Autism in Toddlers-Revised with Follow-up (M-CHAT-R/F)**	Social interest, pretend and functional play, joint-attention, proto-declarative pointing, imitation, motor development, rough and tumble play.	16–30	20	Yes/No	5–10	Parent-reported interview	0–2: low risk 3–7: moderate risk 8–20: high risk	7
**Parent Observation of Early Markers Scale (POEMS)**	Social and communicative development, restricted interests, behavioral and emotional problems.	1–24	61	4-point Likert Scale	30	Parent-reported questionnaire	70	1
**Quantitative CHecklist for Autism in Toddlers (Q- CHAT)**	Social communication, behavior, and language.	18–24	25	5-point Likert scale	5–10	Parent-reported questionnaire	n.s.	2
**Screening for Infants with Developmental Deficits and/or Autism (SEEK)**	I: sleep, eating, and parent-child interaction; II: regulation, parent-child interaction, communication, and coordination stability.	8	SEEK I: 6; SEEK II: 33	Yes/No	3–-40	Parent-reported questionnaire and observational checklist for professionals	n.s.	1
**Three-Item Direct Observation Screen (TIDOS)**	Joint attention, eye contact and response to name.	18–60	3	Yes/no	15–20	Observational checklist for professionals	1	1
**Young Autism and other developmental disorders CHeckup Tool: 18-month-olds’ version (YACHT-18)**	Motor functions, communication and social interaction, pointing, and language comprehension.	18	I: questionnaire (11 items); II: interview (6 questions); II: picture card test.	I: yes/no; III: pass/fail	10	Professionals - reported questionnaire; interview with caregivers, child observation	n.s.	1

Note: n.s. = not specified.

**Table 2:** Details of studies included in the systematic review.

Measure	Author(s) (Year)	Study Design	Population and Subgroups	Application Level (1, 2, or “Hybrid”)	Sens.	Spec.	PPV(NPV)
**ADEC**	[34]	Cross sect.	Study 1 *N* = 19 ASD *N* = 13 ODD *N* = 29 gen pop. Study 2 *N* = 34 PDD *N* = 15 gen. pop. *N* = 5 ODD	hybrid	range: 79%–94%	range: 88%–100%	Study 1: 0.75 (0.90) Study 2: 1 (0.71)
	[35]	Long.	*N* = 55 ASD	Hybrid	100%	89%	0.84 (1 *)
	[36]	Cross sect.	*N* = 70 ASD *N* = 24 PDD-NOS *N* = 57 ODD *N* = 64 gen. pop.	Hybrid	100%	range: 74%–90%	0.84 (1)
	[37]	Cross-sect.	*N* = 96 DC	2	range: 93%–94%	range: 62%–64%	0.83 (0.81)
**AOSI**	[38]	Cross sect.	*N* = 101 GR	2	N/A	N/A	N/A
	[39]	Long.	*N* = 115 GR *N* = 73 DC	2	N/A	N/A	N/A
	[40]	Cross sect.	*N* = 54 GR *N* = 50 DC	2	N/A	N/A	N/A
**BISCUIT**	[41]	Cross-sect.	Study 1 *N* = 957 DC Study 2 *N* = 171 ASD *N* = 144 PDD-NOS	2	Part 1: 84.7%; Part 2: 84.4%; Part 3: 93.4%	Part 1: 86.4%; Part 2: 83.3%; Part 3: 86.6%	N/A
	[42]	Cross-sect.	*N* = 178 ASD *N* = 152 PDD-NOS *N* = 677 gen. pop.	Hybrid	N/A	N/A	N/A
	[43]	Cross-sect.	*N* = 276 DC	2	N/A	N/A	N/A
	[44]	Cross-sect.	Study 1 *N* = 405 ASD Study 2 *N* = 405 ASD *N* = 882 gen. pop.	Hybrid	N/A	N/A	N/A
	[45]	Cross-sect.	*N* = 178 ASD *N* = 152 PDD-NOS *N* = 677 gen. pop.	Hybrid	N/A	N/A	N/A
**CESDD**	[46]	Long.	Wave 1 *N* = 6.808 gen. pop. Wave 2 *N* = 255 at risk Wave 3 *N* = 20 ASD *N* = 40 ODD	1	80%	94%	0.07 (0.99)
**CHAT**	[47]	Cross-sect.	*N* = 50 gen. pop. *N* = 41 GR	2	N/A	N/A	N/A
	[48]	Long.	Wave 1 *N* = 16.000 gen. pop. Wave 2 *N* = 10 ASD *N* = 17 ODD *N* = 23 TD	1	N/A	N/A	N/A
**DBC-ES**	[49]	Cross-sect.	*N* = 142 ASD or PDD *N* = 65 ODD	Hybrid	83%	48%	0.78(0.56)
**ESAT**	[50]	Long.	Wave 1 *N* = 31.724 gen. pop Wave 2 *N* = 255 at risk Wave 3 *N* = 18 ASD *N* = 55 ODD.	1	N/A	N/A	N/A
	[51]	Long.	Wave 1 *N* = 4.107 gen. pop. Wave 2 *N* = 103 at risk	1	N/A	N/A	N/A
**FYI**	[52]	Cross-sect.	*N* = 1300 gen. pop.	1	N/A	N/A	N/A
	[53]	Long.	Wave 1 *N* = 471 gen. pop. Wave 2 *N* = 17 at risk	1	N/A	N/A	N/A
	[54]	Long.	Wave 1 *N* = 699 gen. pop. Wave 2 *N* = 9 ASD	1	N/A	N/A	N/A
**JA-OBS**	[55]	Long.	Wave 1 *N* = 3999 Wave 2 *N* = 64 at risk Wave 3 *N* = 48 ASD *N* = 3 TD *N* = ODD	1	86%	N/A	0.90 (N/A)
**M-CHAT**	[56]	Long.	Wave 1 *N* = 1.122 gen. pop.; Wave 2 *N* = 171 at risk	1	87%	99%	0.80 (0.99)
	[57]	Cross-sect.	*N* = 36 ASD *N* = 18 PDD-NOS *N* = 28 ODD	Hybrid	Critic items: 79%; Total score: 88%	Critic items: 38%; Total score: 38%	0.79(0.28)
	[58]	Cross-sect.	*N* = 122 ASD *N* = 106 gen. pop.	Hybrid	86%	80%	0.81(0.93)
	[59]	Long.	Study 1 Wave 1 *N* = 2480 gen. pop.; Wave 2 *N* = 23 ASD *N* = 63 ODD Study 2: Wave 1 *N* = 2055 gen. pop. Wave 2 *N* = 6 ASD *N* = ODD	1	100%	98%	Study 1: 0.35 (1) Study 2: 0.19 (1)
	[60]	Cross-sect.	*N* = 24 gen. pop. *N* = 25 DC	2	Critic items: 75%; Total score: 65%	Critic items: 89%; Total score: 88%	0.21(0.98)
	[61]	Cross-sect.	*N* = 117 ASD *N* = 339 gen. pop.	Hybrid	N/A	N/A	N/A
	[62]	Cross-sect.	*N* = 141 ASD *N* = 102 ODD	Hybrid	range: 70%–97%	range: 38%–99%	N/A
	[63]	Cross-sect.	*N* = 447 gen. pop.	1			
	[64]	Cross-sect.	*N* = 552 DC	2	range: 70%–97%	range: 38%–99%	N/A
	[65]	Long.	Wave 1 *N* = 51.853 gen. pop. Wave 2 *N* = 173 ASD	1	Critic items: 20.8%; total score: 34.1%	Critic items: 97.9%; total score: 92.7%	Critic items: 0.33 (N/A); total score: 0.15 (N/A)
	[66]	Cross-sect.	*N* = 2048 gen. pop.	1	N/A	N/A	N/A
	[67]	Long.	Wave 1 *N* = 420 gen. pop. Wave 2 *N* = 2 ASD	1	N/A	N/A	N/A
	[68]	Long.	Wave 1 *N* = 1250 DC Wave 2 *N* = 18 ASD *N* = 17 ODD *N* = 1 TD	2	67%	With FUI: 99%; Without FUI: 94%	With FUI: 0.60 (0.99) Without FUI: 0.14 (0.99)
**M-CHAT-R/F**	[69]	Long.	Study 1 *N* = 3309 DC *N* = 484 GR; Study 2 Wave 1: *N* = 1.160 DC *N* = 256 = GR Wave 2 *N* = 80 ASD *N* = 51 ODD	2	N/A	N/A	N/A
	[70]	Cross-sect.	*N* = 207 DC	2	N/A	N/A	N/A
	[71]	Long.	Wave 1 *N* = 16.115 gen. pop. Wave 2 *N* = 123 ASD *N* = 140 ODD	1	94%	83%	0.50 (0.99)
	[72]	Long.	Wave 1 *N* = 2594 gen. pop. Wave 2 *N* = 253 at risk Wave 3 *N* = 17 ASD	1	N/A	N/A	N/A
	[73]	Cross-sect.	*N* = 20 DC *N* = 128 TD	2	N/A	N/A	N/A
	[74]	Long.	Wave 1 *N* = 110 gen. pop. Wave 2 *N* = 18 ASD	1	88.9%	94.6%	0.76 (0.97)
	[75]	Long.	Wave 1 *N* = 7928 gen. pop. Wave 2 *N* = 1140 at risk Wave 3 *N* = 72 ASD	1	96%	86%	0.69 (1)
	[76]	Cross-sect.	*N* = 947 gen. pop.	1	50%	100%	100(0.87)
**POEMS**	[77]	Cross-sect.	*N* = 108 GR	2	74%	73%	0.21 (N/A)
**Q-CHAT**	[78]	Cross sect.	*N* = 779 gen. pop. *N* = 160 ASD	Hybrid	N/A	N/A	N/A
	[79]	Cross-sect.	*N* = 764 gen. pop.	1	N/A	N/A	N/A
	[80]	Cross-sect.	*N* = 139 ASD *N* = 50 PDD *N* = 126 TD	2	73–83%	76–78%	N/A
	[81]	Cross-sect.	*N* = 2400	1	N/A	N/A	N/A
	[82]	Cross-sect.	*N* = 545	1	N/A	N/A	N/A
**SEEK**	[83]	Cross-sect.	*N* = 312 gen. pop.	1	N/A	N/A	N/A
**TIDOS**	[84]	Cross-sect.	*N* = 86 ASD *N* = 76 ODD *N* = 97 gen. pop.	Hybrid	95%	85%	0.91(0.90)
**YACHT-18**	[85]	Cross-sect.	*N* = 2.814 gen. pop.	1	60%	86.3%	N/A

Note: Cross-sect. = Cross-sectional study; Long. = longitudinal study; ASD = children with ASD; gen. pop. = general population; ODD = other developmental disorders; PDD = Pervasive Developmental Disorder; PDD-NOS = pervasive developmental disorder—not otherwise specified; TD typically developing children; “hybrid” level of application = level 1 and 2 screening measure applied to other population (e.g., clinical sample); FUI: follow-up interview; Sens = sensitivity; Spec = specificity; PPV(NPV) = Positive Predictive Value (Negative Predictive Value); N/A = not available. * Authors reported PPV and NPV values from [36] study.

**Table 3:** Results of the COSMIN evaluation.

Measures	*Author(s), (Years)*	*Psychometric Properties*								
		Internal Consistency	Reliability	Measurement Error	Content Validity	Structural Validity	Hypothesis Testing	Cross-Cultural Validity	Criterion Validity	Responsiveness
**ADEC**	[34]	0 *unidimensionality, sample*	+ *missing item*, *sample, time interval*				0 *sample*	0 *sample*	0 *sample*	
	[35]						+ *missing item*		+ *missing item*	
	[36]	0 *unidimensionality*	0 *time interval*			+ *missing item*	+ *missing item*		+ *missing item*	
	[37]	0 *unidimensionality*					+ *missing item, hypothesis*		+ *missing item*	
**AOSI**	[38]		+ *sample*, *missing item*		+++					
	[39]						+ *missing item*		+ *missing item*	
	[40]			+ *missing item*			+ *missing item*		+ *missing item*	+ *missing item*
**BISCUIT**	[41]						0 *comparator instrument*		0 *statistical methods*	
	[42]						+ *missing item, hypothesis, comparator instrument*		+ *missing item*	
	[43]	0 *unidimensionality*								
	[44]	+ *missing item*			+++	+ *missing item*	+ *missing item*		0 *statistical method*	
	[45]	0 *unidimensionality*					+ *missing item, hypothesis*		0 *no gold standard*	
**CESDD**	[46]						+ *hypothesis*		+ *missing item*	
**CHAT**	[47]		+++		+ ++		0 *comparator instrument*		0 *no gold standard*	
	[48]						+ *missing item*		+ *missing item*	
**DBC-ES**	[49]	0 *unidimensionality*	0 *only one measurement*				+ *missing item, hypothesis*		+ *missing item*	
**ESAT**	[50]			*0 time interval*			+ *missing item, hypothesis*		+ *missing item*	
	[51]						+ *missing item*		+ *missing item*	
**FYI**	[52]	+++			+++	0 *statistical method*	++		+++	
	[53]	0 *statistical method*					++	0 *statistical method*	++	
	[54]			0 *time interval, measurement condition*			+ *sample*		+ *missing item, sample*	+ *missing item, statistical method*
**JA-OBS**	[55]		0 *only one measurement*						+ *missing item*	
**M-CHAT**	[56]				+++	.	+ *missing item, sample*		0 *missing item*	
	[57]	0 *unidimensionality*					++		++	
	[58]							+ *missing item*		
	[59]						+ *missing item, hypothesis*	0 *missing item, translation*	+ *missing item*	
	[60]	0 *sample, unidimensionality*	0 *sample*				0 *sample, hypothesis*		0 *sample, comparator instrument*	
	[61]	0 *unidimensionality*					+ *missing item, hypothesis*		+ *missing item*	
	[62]						+++		0 *statistical method*	
	[63]	0 *measurement not independent*					0 *comparator instrument*			
	[64]		0 *only one measurement*				0 *comparison instrument*			
	[65]			+ *time interval*			+ *sample, hypothesis*		0 *missing item*	
	[66]					+++				
	[67]		+ *missing item*					0 *statistical method*		
	[68]						0 *missing item, hypothesis*		0 *missing item, sample*	
**M-CHAT-R/F**	[69]	0 *unidimensionality*		0 *measurement not indipendent*			+ *missing item*		0 *statistical method*	0 *statistical method*
	[70]								+ *missing item*	
	[71]	0 *unidimensionality*	0 *administration not similar, statistical method*				+ *hypothesis*		+++	
	[72]	0 *sample, unidimensionality*	0 *time interval, statistical method*				++	0 *expertise translator, statistcal method*	0 *sample*	
	[73]	0 *sample, undimensionality*	0 *missing, sample, time interval, statistical method*					0 *statistical method*		
	[74]		+ *missing item*				0 *comparator instrument*	+ *translation*	0 *no golden standard*	
	[75]	0 *undimensionality*	0 *statistical method*				0 *hypothesis*	0 *no pilot study, statistical method*	+++	
	[76]								0 comparator instrument	
**POEMS**	[77]	0 *unidimensionality*	++		+++		0 *hypothesis*		0 *statistical methods*	
**Q-CHAT**	[78]	+++	+ *measurement condition*		+++		+ *hypothesis*			
	[79]	+++	0 *time interval*			+++	+ *hypothesis*		0 *comparator instrument*	
	[80]	+++					0 comparator instrument		+++	
	[81]	+++				+++	0 comparator instrument	0 statistical method		
	[82]	+ missing item				+ missing item	+ missing item	0 no pilot study; statistical method		
**SEEK**	[83]				+++					
**TIDOS**	[84]				+++		+ *missing item*		+ *missing item*	
**YACHT- 18**	[85]								+ *missing item*	

Note: 4-point scale rating: +++ = excellent, ++ = good, + = fair, 0 = poor. Empty cell = COSMIN rating not evaluated. Ratings fair and poor were explained with the reason(s) in italics leading the evaluation. Specifically, “administration not similar” means the two administration conditions to examine measure property were not similar; “comparator instrument” means that authors did not administered a gold standard measure for ASD to evaluate the criterion validity;“expertise translator” means that the expertise of measure translators was poor or not described by authors; “hypothesis” means that the authors did not formulate the hypothesis a priori; “missing item” means that the authors did not report the percentage and/or the handling method for missing data; “no pilot study” means the translated measure did not pre-tested in a target population; “only one measurement” means the authors did not administered the measure at least two times; “sample” means that the sample size was not adequate;“statistical method” means that authors did not calculated the right parameter(s) for the specifc property;“time interval” means that the time interval between two measurements was not adequate;“translation” means that the back-translation process was not adequately described; “unidimensionality” means that the internal consistency parameter was not calculated for each (sub)scale separately [29,30].
